# Increased operative time is an independent risk factor for surgical complications following isolated anterior cruciate ligament reconstruction in skeletally immature patients

**DOI:** 10.1002/jeo2.70759

**Published:** 2026-05-19

**Authors:** Rishi Sinha, Robert Van Pelt, Daniel W. Green, Kevin G. Shea, Emily L. Niu, Michael G. Saper, Matthew R. Schmitz, Theodore J. Ganley, Philip L. Wilson, Henry B. Ellis, SCORE Quality Improvement Registry, Jay Albright, Sheila Algan, Jennifer Beck, Richard Bowen, Jennifer Brey, Matthew Brown, Marc Cardelia, Charles Chan, Christian Clark, Allison Crepeau, Eric Edmonds, Matthew Ellington, Henry Ellis, Peter Fabricant, Jeremy Frank, Ted Ganley, Dan Green, Benton E. Heyworth, Kevin Latz, Todd Lawrence, Alfred Mansour, Stephanie Mayer, Scott McKay, Matt Milewski, Emily Niu, Donna Pacicca, Shital Parikh, Jason Rhodes, Stephanine Pearce, Michael Saper, John Schlechter, Greg Schmale, Matt Schmitz, Kevin Shea, Stephen Storer, Curtis VandenBerg, Phil Wilson, Yi‐Meng Yen

**Affiliations:** ^1^ Carilion Clinic Department of Orthopaedic Surgery Roanoke Virginia USA; ^2^ Virginia Tech Carilion School of Medicine Roanoke Virginia USA; ^3^ Scottish Rite for Children Dallas Texas USA; ^4^ University of Texas Southwestern Medical Center Dallas Texas USA; ^5^ Department of Orthopedic Surgery Hospital for Special Surgery New York New York USA; ^6^ Department of Orthopedic Surgery Stanford University Palo Alto California USA; ^7^ Department of Orthopedic Surgery and Sports Medicine Children's National Hospital Washington DC USA; ^8^ Department of Orthopedics and Sports Medicine Seattle Children's Hospital Seattle Washington USA; ^9^ Division of Orthopaedic Surgery Rady Children's Hospital San Diego California USA; ^10^ Children's Hospital of Philadelphia, Sports Medicine and Performance Center Philadelphia Pennsylvania USA; ^11^ Children's Hospital Colorado Aurora Colorado USA; ^12^ Oklahoma Children's Hospital Oklahoma City Oklahoma USA; ^13^ Boulder Community Health Boulder Colorado USA; ^14^ Department of Orthopaedic Surgery, David Geffen School of Medicine at UCLA Los Angeles California USA; ^15^ Orthopedic Institute for Children Los Angeles California USA; ^16^ Norton Children's Hospital of Louisville Louisville Kentucky USA; ^17^ Connecticut Children's Hospital Hartford Connecticut USA; ^18^ Children's Hospital of the King's Daughters Norfolk Virginia USA; ^19^ Stanford University Orthopaedics Palo Alto California USA; ^20^ OrthoCarolina Pediatric Orthopaedic Center Charlotte North Carolina USA; ^21^ Rady Children's Hospital San Diego California USA; ^22^ Central Texas Pediatric Orthopedics & Scoliosis Surgery, Dell Medical School The University of Texas at Austin Austin Texas USA; ^23^ Scottish Rite for Children, University of Texas Southwestern Medical Center Dallas Texas USA; ^24^ Hospital for Special Surgery, Weill Cornell Medical College New York City New York USA; ^25^ Joe DiMaggio Children's Hospital Hollywood Florida USA; ^26^ Children's Hospital of Philadelphia Philadelphia Pennsylvania USA; ^27^ Hospital for Special Surgery New York New York USA; ^28^ Boston Children's Hospital Boston Massachusetts USA; ^29^ Children's Mercy Kansas City Missouri USA; ^30^ UTHealth Houston, McGovern Medical School Houston Texas USA; ^31^ Texas Children's Hospital Houston Texas USA; ^32^ Children's National Medical Center Washington DC USA; ^33^ Cincinnati Children's Hospital Cincinnati Ohio USA; ^34^ Nemours Children's Health Wilmington Deleware USA; ^35^ Seattle Children's Hospital Seattle Washington USA; ^36^ Children's Hospital of Orange County Orange California USA; ^37^ Boston's Children Hospital Boston Massachussets USA

**Keywords:** adolescent, anterior cruciate ligament, complications, operative time, surgical site infection

## Abstract

**Purpose:**

In the context of increasing intensive year‐round training amongst youth athletes, the incidence of paediatric anterior cruciate ligament (ACL) reconstruction has risen markedly. There is a growing interest in minimising surgical complications for these patients. Although the literature has demonstrated an association between operative time and complications for ACL reconstruction in adults, this has not been explored in younger patients. The primary purpose of this study was to determine whether operative time is a risk factor for complications following ACL reconstruction in skeletally immature patients. Secondary aims included identifying the most common types of complications in this cohort and identifying which of these complications were associated with increased operative time.

**Methods:**

A prospective, multicenter surgeon‐driven registry was reviewed to identify skeletally immature patients who underwent primary isolated ACL reconstruction, with a minimum 8‐month follow‐up. Demographics, surgical characteristics, operative time and complications were recorded. Continuous variables were compared via an independent *t*‐test or analysis of variance, and categorical variables were compared via Chi‐squared or Fisher's exact tests. Comparisons were deemed to be statistically significant using a threshold of *p* < 0.05. Multiple logistic regression analysis was performed to control for baseline characteristics.

**Results:**

A total of 719 patients were included with a mean follow‐up of 17.6 ± 8.6 months (range 8–30 months), 483 (67.2%) were male. Mean age was 13.3 ± 1.9 years. Mean operative time was 115.1 ± 44.2 min. The complication rate for the cohort was 15.3%. After adjusting for baseline characteristics, increased operative time remained an independent risk factor for developing a complication (OR = 1.19, *p* = 0.002). Specifically, increased operative time was an independent risk factor for surgical site infection (OR = 1.30, *p* = 0.019) and arthrofibrosis (OR = 1.36, *p* = 0.009).

**Conclusions:**

Increased operative time was found to be an independent risk factor for complications following ACL reconstruction for skeletally immature patients. Specifically, operative time was associated with surgical site infection and arthrofibrosis. Future efforts to improve operative efficiency are warranted to improve patient outcomes.

**Level of Evidence:**

Level III.

**Abbreviations**
ACL: anterior cruciate ligament
ACLR: ACL reconstruction
BMI: body mass index
CDC: Centers for Disease Control and Prevention
HIPAA: Health Insurance Portability and Accountability Act
ITB: iliotibial band
LET: lateral extra‐articular tenodesis
SCORE: Sports Cohort Outcomes Registry
SD: standard deviation
US: United States

## INTRODUCTION

Injuries to the anterior cruciate ligament (ACL) have become increasingly common amongst youth athletes with an increase of ACL reconstructions performed in skeletally immature patients [5, 7, 11, 41, 56]. Many paediatric orthopaedic and sports medicine physicians advocate for early operative management of ACL injuries in this population due to concerns of recurrent instability and chondral and meniscal injury that may lead to early osteoarthritis with nonoperative management [14, 31]. There is a growing interest in identifying and minimising postoperative complications for these patients to improve their outcomes. Common complications following ACL reconstruction for these patients include, but are not limited to, arthrofibrosis, surgical site infections, growth disturbance and graft failure [15, 16, 58]. Reinjury and graft failure have been extensively studied and several potential risk factors identified, including younger patient age, female gender, ligamentous laxity, knee recurvatum, excess posterior tibial slope and coronal plane deformities [32, 35, 36, 37, 42, 47]. Many of these factors are inherent to the patient and thus either nonmodifiable or can only be addressed via additional procedures. Specific risk factors for other common complications following ACL reconstruction (ACLR) are less established. One modifiable variable that may affect complication risk in this population is operative time. A longer operative time has been well‐documented as a risk factor for complications following a variety of adult orthopaedic procedures, including adult ACL reconstruction [6, 13, 21, 22, 25, 33, 43, 51]. However, this association has not been studied for ACL reconstruction in skeletally immature patients.

The primary purpose of this study was to determine whether operative time is an independent risk factor for developing complications following ACL reconstruction in skeletally immature patients. In addition, we aimed to identify the most frequent complications and to determine which of them were associated with prolonged operative time.

## METHODS

### Study design and study population

This was a retrospective analysis of a consecutive series of patients entered in the Sports Cohort Outcomes Registry (SCORE). This is a prospective, multicenter surgeon‐driven quality improvement registry that includes 41 paediatric and/or sports medicine fellowship‐trained surgeons from 25 institutions. The registry database utilises a Health Insurance Portability and Accountability ACT (HIPAA)‐compliant platform (Scribe System; Web Data Solutions), which contains no identifiable patient data. Data quality is maintained using a biannual audit to ensure the accuracy of the collected data. This study was IRB‐exempt at our institution.

Consecutive paediatric patients treated with ACL reconstruction from 2018 to 2023 were identified using a review of the SCORE database. Skeletally immature patients, as defined by the presence of an ‘open’ or ‘closing’ knee physis on preoperative magnetic resonance imaging, who underwent a primary isolated ACL reconstruction with a minimum of 8‐month follow‐up were included. Based on the follow‐up times set by the SCORE registry, the 8‐month follow‐up time was chosen to maximise the availability of data for analysis while capturing most of the major postoperative complications. Excluded were skeletally mature patients, as defined by the presence of a ‘closed’ physis, patients who had any concomitant procedure (e.g., meniscal repair) at the time of surgery, patients for whom the ACL reconstruction was a revision surgery, and patients with less than 8‐month follow‐up.

Patient demographic data collected included age, gender and body mass index (BMI) percentile by age. Surgeon characteristics included surgeon experience and the type of surgical assistant present during the case. Operative characteristics collected included operative time, type of anaesthesia, use of a tourniquet and ACL graft technique. Surgeon experience was defined as the number of years in practice following fellowship completion and was divided into categories of 0–10 years and greater than 10 years. BMI percentile by age was categorised according to the Center for Disease Control (CDC) guidelines as underweight (less than the 5th percentile), healthy weight (5th percentile to less than 85th percentile), overweight (85th percentile to less than 95th percentile) or obese (greater than or equal to 95th percentile). The type of surgical assistant present was categorised into attending surgeon partner, fellow ± resident or midlevel, resident + midlevel, resident only, midlevel only or none. The term ‘midlevel’ refers to an advanced practice provider in the field of orthopaedics (for instance, a physician assistant or certified nurse practitioner). The type of anaesthesia used was categorised into regional anaesthesia only, or general anaesthesia (with or without regional anaesthesia). ACL graft technique was categorised as transphyseal, all epiphyseal, iliotibial band (ITB) extraosseous extraphyseal and combined transphyseal and ITB lateral extra‐articular tenodesis (LET). The specific ACL graft technique used was based on the discretion of the individual treating surgeon.

Operative time was defined as the time from skin incision to skin closure. This was recorded and collected as a continuous variable. During analysis, operative time was divided into 15‐min increments to determine marginal increases in risk of complications associated with prolonging the operative time by 15 min.

Complications occurring during the duration of a patient's follow‐up after surgery were recorded for each patient. The most common complications in the cohort were identified. The total rate of complications, and the rate of each of the most common complications, was calculated for the cohort.

### Statistical analysis

Descriptive analyses were performed to describe the study population, and univariate analysis was initially performed to determine the association between operative time and the risk of surgical complications. The association of baseline patient and perioperative characteristics with operative duration was initially analysed using bivariate linear regression. Subsequently, multivariate logistic regression was performed to determine which of these baseline characteristics was associated with operative time when controlling for all other variables. The association between operative time and the risk of complications was also initially analysed using bivariate linear regression. Testing for model linearity revealed no significant deviation from linearity in the association between operative time and complications (*p* = 0.069). Subsequent multivariate logistic regression was performed to determine the association between operative time and risk of complications when controlling for all baseline patient and perioperative characteristics. Fifteen‐minute increments in operative time were used to allow for clinical interpretation of the impact of finite increases in operative time on the risk of developing surgical complications. Statistical significance was defined using a threshold of *p* < 0.05. Analyses were performed with SAS 9.4 (SAS Institute). Given the retrospective study design, a post hoc power analysis would be the only option to assess for power. This was deferred due to the inherent limitations of post hoc power analyses in accurately determining power [2, 20, 27].

## RESULTS

### Baseline demographic and procedure characteristics

**Figure 1:**
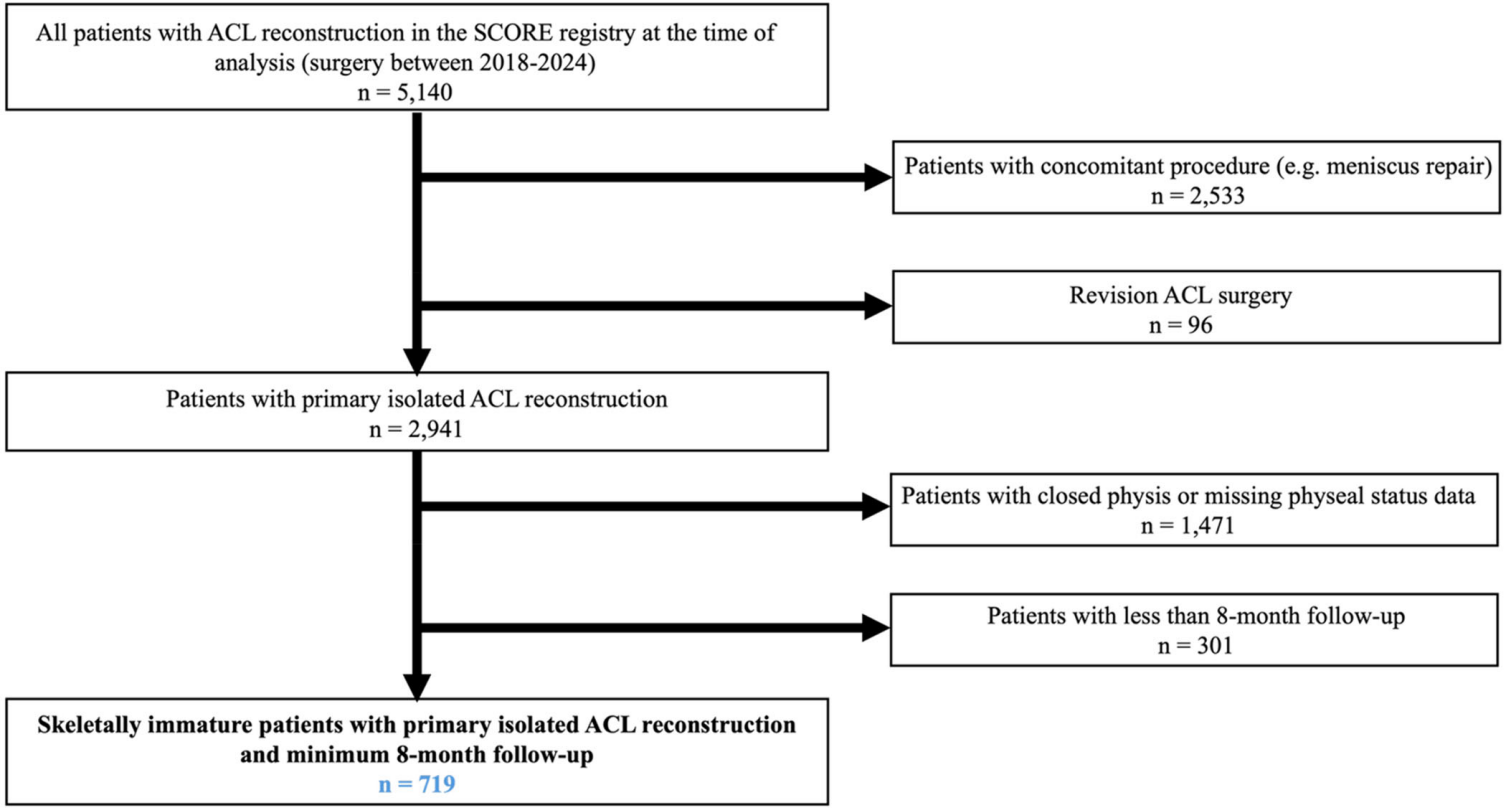
Flowchart of patient inclusion and exclusion. ACL, anterior cruciate ligament; SCORE, Sports Cohort Outcomes Registry.

At the time of analysis, 719 skeletally immature patients who underwent primary isolated ACL reconstruction with a minimum 8‐month follow‐up were included in the study (Figure 1). A total of 483 (67.2%) patients were male. Mean age at time of surgery was 13.3 ± 1.9 years (range, 5.0–19.0 years), and mean follow‐up was 17.6 ± 8.6 months (range, 8.0–30.0 months). The mean BMI percentile by age was 65.5 ± 21.7 (range, 0.3–99.9) (Table 1).

**Table 1:** Baseline characteristics of the overall cohort (*n* = 719 patients).

Characteristic		*n* (%)
Gender	Male	483 (67.2%)
	Female	236 (32.8%)
Age at surgery (years)		13.3 ± 1.9
BMI percentile by age		65.5 ± 21.7
Surgeon years in practice	0–10	174 (24.2%)
	>10	545 (75.8%)
Surgical assistant present	Attending surgeon partner	25 (3.5%)
	Fellow ± resident or midlevel	139 (19.7%)
	Resident + midlevel	91 (12.9%)
	Resident only	174 (24.3%)
	Midlevel only	242 (33.7%)
	None	42 (5.9%)
Anaesthesia type	Regional only	57 (7.9%)
	General^a^	662 (92.1%)
Tourniquet use	Yes	565 (78.6%)
	No	154 (21.4%)
ACL graft technique	Transphyseal	331 (46.0%)
	All‐epiphyseal^b^	116 (16.1%)
	ITB extraosseous extraphyseal	164 (22.8%)
	Combined transphyseal and ITB LET	108 (15.0%)
Operative time (minutes)		115.1 ± 44.2
Follow‐up time (months)		17.6 ± 8.6

Abbreviations: ACL, anterior cruciate ligament; BMI, body mass index; ITB, iliotibial band; LET, lateral extra‐articular tenodesis.

^a^ Includes any case where general anaesthesia was used (including cases where regional was also used).

^b^ Includes 87 cases of all‐epiphyseal femoral with all‐epiphyseal tibial technique; and 29 cases of all‐epiphyseal femoral with transphyseal tibial technique.

These 719 patients were treated by 32 different surgeons. Of the 719 patients, 545 (75.8%) were treated by surgeons with greater than 10 years of experience. This resulted in a mean case number per surgeon for the study of 22.5 cases amongst all surgeons, and 22.4 cases amongst surgeons with greater than 10 years of experience. During the case, the most common type of surgical assistant present in the operating room was midlevel only (33.7%), followed by a resident only (24.3%) and a fellow ± resident or midlevel (19.7%). Most surgeries involved the use of general anaesthesia (92.1%) and tourniquet (78.6%), and most surgeries were performed using a transphyseal technique (46.0%) (Table 1).

### Operative time by demographic and procedure characteristics

The mean operative time of all surgeries performed in the cohort was 115.1 ± 44.2 min (range, 30–225 min) (Table 1). When controlling for all baseline demographic and perioperative characteristics, multivariate analysis revealed that operative times were significantly increased in patients who were obese (125.0 ± 40.6 min, *p* < 0.001), patients who received regional only anaesthesia during the case (134.3 ± 35.3 min), and patients who did not have a tourniquet placed during surgery (128.8 ± 33.7 min) (Table 2). Additionally, cases in which an attending surgeon partner was present as an assistant in the operating room had a significantly shorter operative time on average (94.1 ± 35.7 min, *p* = 0.014) (Table 2).

**Table 2:** Operative time by demographic and procedure characteristics (*n* = 711).

	*n*	Operative time (minutes)	Multivariate^a^	
			RR (95% CI)	*p*‐Value
Gender				0.601
Male	483	111.2 ± 35.4	Reference	
Female	236	114.7 ± 37.8	1.02 (0.95 – 1.10)	
BMI category^b^				
Underweight	16	95.6 ± 26.3	0.81 (0.60–1.07)	
Healthy weight	431	109.0 ± 34.6	Reference	
Overweight	139	113.0 ± 36.4	1.05 (0.96–1.14)	
Obese	121	125.0 ± 40.6	1.22 (1.12– 1.34)	**<0.001**
Surgeon years in practice				
0–10	174	117.3 ± 37.7	Reference	
>10	545	110.7 ± 35.7	1.04 (0.96–1.13)	0.292
Surgical assistant present				
Attending surgeon partner	25	94.1 ± 35.7	0.72 (0.56 – 0.93)	**0.014**
Fellow ± resident or midlevel	139	115.0 ± 35.8	0.99 (0.56–0.93)	
Resident + Midlevel	91	116.0 ± 33.6	1.02 (0.85–1.21)	
Resident only	174	112.0 ± 42.6	0.92 (0.78–1.09)	
Midlevel only	242	111.0 ± 33.4	0.98 (0.84–1.15)	
None	42	112.0 ± 30.6	Reference	
Anaesthesia type				
Regional only	57	134.6 ± 35.7	1.55 (1.26–1.77)	**<0.001**
General	662	110.2 ± 35.6	Reference	
Tourniquet use				
Yes	565	107.7 ± 35.5	Reference	
No	154	128.8 ± 33.7	1.37 (1.25–1.50)	**<0.001**
ACL graft technique				
Transphyseal	331	111.1 ± 34.2	Reference	
All‐epiphyseal	116	112.0 ± 40.7	1.03 (0.93–1.13)	
ITB extraosseous extraphyseal	164	110.9 ± 28.7	0.89 (0.80–0.98)	
Hybrid combined transphyseal and ITB extraosseous	108	114.0 ± 33.3	1.01 (0.90–1.12)	0.193

*Note*: The bold values indicate the relationships that were found to be statistically significant (i.e., a *p* value that is less than 0.05).

Abbreviations: ACL, anterior cruciate ligament; BMI, body mass index; CI, confidence interval; ITB, iliotibial band; RR, risk ratio.

^a^ Adjusted for all characteristics in this table.

^b^ Based on Centers for Disease Control and Prevention (CDC) guidelines for BMI percentile by age for children and teenagers.

### Association between operative time and complications

**Figure 2:**
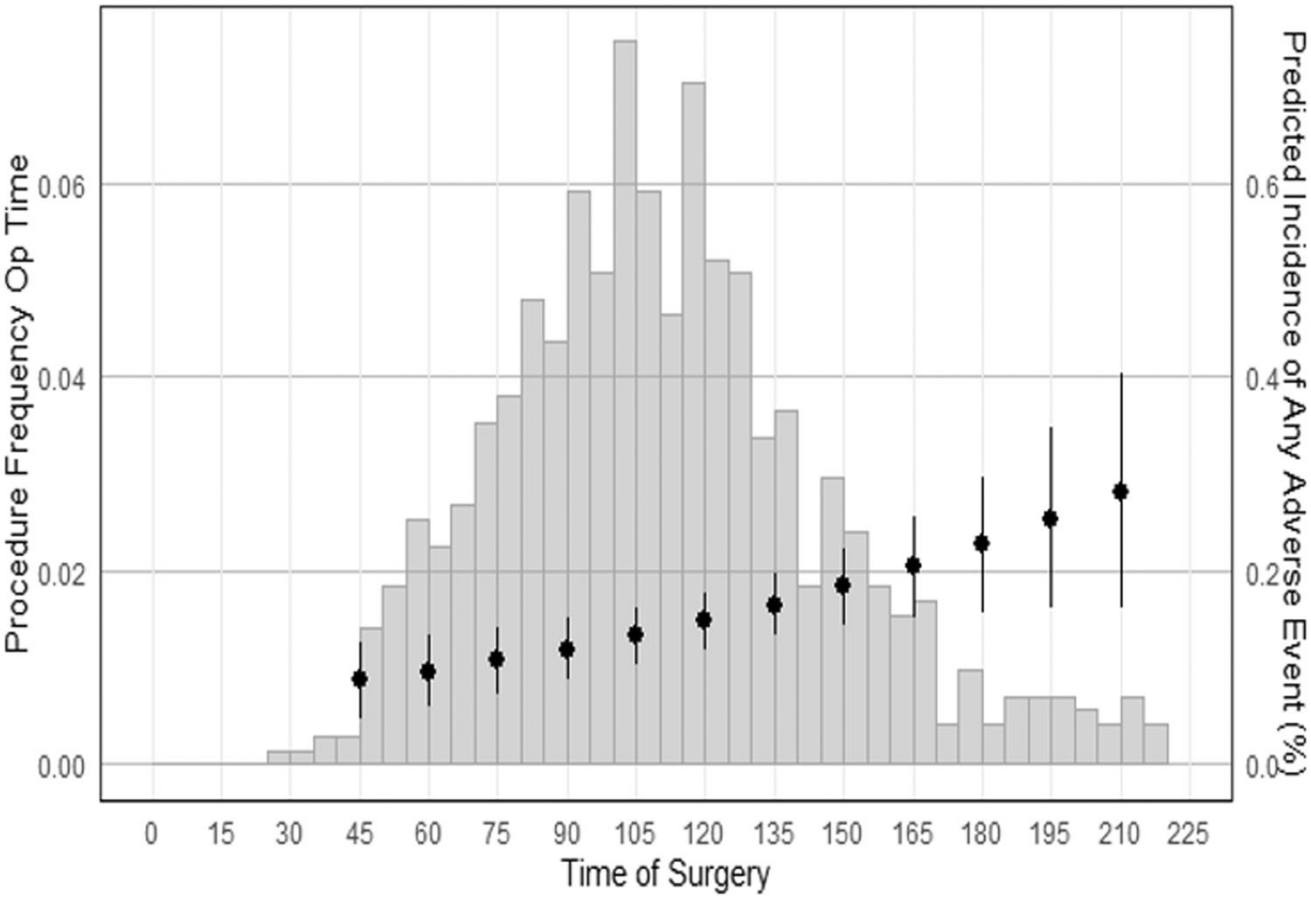
Predicted linear correlation between operative time and complications.

The complication rate for the overall cohort was 15.3%, with the most common complications including graft failure (4.73%), arthrofibrosis (2.92%), surgical site infection (2.36%) and recurrent/persistent/uncontrolled pain (2.23%) (Table 3). Univariate analysis revealed that patients who experienced a complication following their surgery had on average an approximately 14‐min longer operative time versus patients who did not experience a complication (124.3 ± 41.1 vs. 110.3 ± 51.1 min, *p* = 0.02). After adjusting for all demographic and procedural characteristics listed in Table 2, multivariate analysis revealed that increased operative time by 15‐min was associated with an increased overall risk of developing a complication (OR = 1.19, *p* = 0.002) (Table 3, Figure 2). When analysing for specific complications, increased operative time by 15‐min was found to be associated with an increased risk of surgical site infection (OR = 1.30, *p* = 0.019) and an increased risk of arthrofibrosis (OR = 1.36, *p* = 0.009) (Table 3). Arthrofibrosis was defined based on prior studies as loss of extension ≥10° and/or loss of knee flexion ≥25° compared to the contralateral side at least 3 months following surgery [18, 46, 55].

**Table 3:** Association of 15‐min increase in operative time with rate of complications.

	Rate (%)	Multivariate^a^	
		OR (95% CI)	*p*‐Value
**Complication**	**15.29**	**1.19 (1.06−1.27)**	**0.002**
**Surgical site infection** b	**2.36**	**1.30 (1.00−1.53)**	**0.019**
Hospital readmission	0.28	1.30 (1.13–1.50)	0.996
Reoperation^c^	0.42	1.63 (1.03–2.92)	0.119
DVT or PE	0.14	0.82 (0.20–3.99)	0.407
Sensory or motor loss	0.42	0.80 (0.22–2.14)	0.774
**Arthrofibrosis**	**2.92**	**1.36 (1.08–1.58)**	**0.009**
Recurrent/persistent/uncontrolled pain	2.11	1.00 (0.79–1.24)	0.987
Hemarthrosis or effusion	0.56	0.76 (0.39–1.22)	0.156
Haematoma or seroma	0.28	1.95 (0.94–6.01)	0.119
Dermatologic complaint (rash, skin ulcer, blister)	0.56	1.10 (0.74–1.58)	0.605
Graft failure	4.73	1.04 (0.89–1.20)	0.184
Continued mechanical symptoms or instability	0.42	1.26 (0.76–2.00)	0.891
Acquired leg length deformity	0.28	1.06 (0.58–1.76)	0.818
Acquired angular deformity	0.42	0.57 (0.08−1.82)	0.996

*Note*: The bold values indicate the relationships that were found to be statistically significant (i.e., a *p* value that is less than 0.05).

Abbreviations: CI, confidence interval; DVT, deep vein thrombosis; OR, odds ratio; PE, pulmonary embolism.

^a^ Adjusted for all demographic and p.rocedure characteristics listed in Table 1.

^b^ Includes both superficial and deep surgical site infection.

^c^ Excludes revision surgery for graft failure.

## DISCUSSION

Our review of a prospective, multicenter surgeon‐driven quality improvement registry revealed that when controlling for demographic and perioperative characteristics, prolonged operative time by 15‐min was associated with an increased risk of developing surgical complications following isolated ACL reconstruction for skeletally immature patients. Specifically, increased operative time was associated with an increased risk of surgical site infection and arthrofibrosis. With an average operative time for the entire cohort of 115 min, patients who sustained a complication had on average a 14 min longer surgical time compared to those patients who did not sustain a complication. Though some risk factors for postoperative complications are intrinsic to the patient and may be nonmodifiable, operative time is a potentially modifiable variable. Surgeons should strive to identify ways to minimise operative time to reduce the risk of complications and improve outcomes in this population.

Increased operative time has previously been shown to be associated with postoperative complications in adult orthopaedic patients [13, 21, 22, 25, 43, 51]. Agarwalla et al. demonstrated that in adults undergoing ACL reconstruction, increased operative time was independently associated with an increased risk of complications [1]. Our results are consistent with these findings and are the first study, to our knowledge, to investigate the association between operative time and complications in skeletally immature paediatric patients undergoing ACL reconstruction. Unlike most of the adult orthopaedic literature, our study was not limited to short‐term complications within 30 or 90 days, and instead included an average length of follow‐up of almost 18 months. This allowed us to identify longer‐term complications, facilitating a more accurate assessment of the impact of operative time on the true complication rate following surgery.

Surgical site infections investigated in our study included both superficial infections, which are defined by the CDC as limited to the skin and subcutaneous tissue at the site of incision, and deep infections, which are defined as involving fascial and muscle layers [29]. An increased duration of surgery leaves the surgical site exposed for a greater amount of time, potentially increasing the risk for pathologic organisms to seed the wound. Additionally, increased duration of anaesthesia can increase the risk of intraoperative hypothermia, which may make the patient more susceptible to infection [10, 12, 19]. Given that surgical site infection is a major risk factor for increased morbidity and increased economic burden to both patient families and hospitals [3, 48, 57], targeting operative time to potentially reduce the risk of surgical site infection in this population is warranted.

Arthrofibrosis is a potentially devastating complication of ACL reconstruction, often resulting in significant functional impairment and decreased patient satisfaction, with the potential need for manipulation under anaesthesia and surgical lysis of adhesions to regain motion [17, 30, 39]. Previously reported risk factors for arthrofibrosis following paediatric ACL reconstruction include increased BMI, female gender and patellar tendon autograft [40, 44, 45]. Our study indicates that increased operative time may also be a risk factor for the development of arthrofibrosis in these patients. Given that the pathogenesis of arthrofibrosis may involve an inflammatory reaction to an insult such as surgery [53], it is conceivable that a prolonged duration of surgery may be associated with increased inflammation leading to arthrofibrosis. Future research is needed to better understand this association.

This investigation also identified various patient and perioperative characteristics associated with increased operative time, including obesity and lack of tourniquet use. Our finding of increased operative time in obese patients is similar to findings previously reported for obese adult patients undergoing ACL reconstruction [9] and elective rotator cuff repair [54]. There is a paucity of literature on tourniquet use in paediatric ACL patients, and in adult ACL reconstruction, its use remains controversial and subject to surgeon preference. The reduction of blood flow to the lower limb with tourniquet application is thought to improve visualisation of the surgical field [28, 34], and a recent randomised control trial of adult ACL reconstructions found tourniquet use to be associated with decreased operative time [59]. However, some studies have failed to demonstrate this association [50]. Our study is the first, to our knowledge, to investigate the effects of tourniquet use in paediatric ACL reconstruction, and future work is warranted to support our findings of increased operative time without tourniquet application in these patients.

Additionally, our investigation identified the presence of an attending surgeon partner as the surgical assistant in the operating room to be associated with decreased operative time. This association is intuitive in the sense that an attending surgeon partner who has completed their training and has experience working with the primary surgeon would facilitate a quicker surgery. Importantly, the addition of a resident or fellow did not increase operative time versus performing the surgery without an assistant present. Additionally, there was no difference in operative time based on the level of trainee (fellow vs. resident), and there was no difference in operative time with the presence of resident or fellow versus the presence of midlevel. These findings are consistent with prior studies in paediatric orthopaedics and add to the growing body of evidence that a lower level of experience of a surgical trainee assisting in the operating room does not negatively impact patient care [4, 26, 52]. This is important in the context of academic orthopaedics, where surgeons must balance their commitment to patient care with their responsibility to train future surgeons.

The association between prolonged operative time and complications demonstrated in this study raises questions about potential interventions to reduce operative time and, therefore, improve patient outcomes. One potential strategy utilises surgeon coaching programs to help providers improve their operative efficiency. Surgical coaching programs, consisting of peer‐to‐peer and video‐assisted coaching designed to improve surgeon performance, have been shown to have a high perceived value for professional development and to promote increased teamwork, communication and awareness in the operating room [23, 24, 38, 49]. Additionally, recent studies have demonstrated coaching programs within general and bariatric surgery to be effective in reducing operative time at both the chief resident and the attending physician level [8, 23]. Future efforts to incorporate surgeon coaching for paediatric orthopaedic procedures, including ACL reconstruction, may prove to be effective in reducing operative time and thereby reducing complication rates. Efforts should also be made to identify individual steps or components of surgery that could be improved to ensure a shorter operative time. While our study identified tourniquet use as a potential way to reduce operative time, a more in‐depth examination into the different components and steps of ACL reconstruction that could be targeted to shorten operative duration is warranted.

While this study was a retrospective review of a prospective, multi‐surgeon quality improvement registry, there are several important limitations to consider. There are inherent limitations to using large database studies, including reporting errors and sampling bias. These types of studies operate under the assumption that all data was recorded and coded accurately. Although this remains a limitation, rigorous biannual audits are performed for the SCORE database, and an additional quality check was performed for this study specifically, to verify the accuracy of the collected data. An additional limitation is that cases where regional‐only anaesthesia was used occurred primarily at a single centre. Therefore, the difference in operative time seen in our study with regional‐only anaesthesia may be due to other factors inherent to that single centre that were not accounted for. Additionally, while this study sought to control for important patient and perioperative characteristics, it is nonetheless difficult to establish causation. Other factors may be associated with operative time, such as additional trauma or swelling during the procedure or inherent complexity of the surgery, and these factors may also be associated with increased risk of complications. These types of variables were not collected and accounted for during the study, making it difficult to establish a true cause‐and‐effect relationship between operative time and complication rate. It is possible that these other variables are causative of the increased risk of complications directly, not just indirectly via increased operative time. A final limitation is that our study excludes patients who had concomitant injuries and surgeries performed. While this may limit the generalisability of our findings, isolated ACL reconstruction remains a commonly performed procedure in this patient population. Excluding cases with concomitant procedures reduced the potential for bias based on the type of procedure that was performed. Though outside the scope of the present study, future research may investigate whether our findings are generalisable to patients who undergo additional procedures in addition to the ACL reconstruction.

## CONCLUSIONS

In conclusion, our study found operative time to be independently associated with complications, specifically surgical site infection and arthrofibrosis, following ACL reconstruction in skeletally immature patients. Given the potential severity of these complications, exploring methods to reduce operative time is warranted. Future research investigating strategies such as surgeon coaching programs to decrease operative time should be pursued in order to improve patient outcomes.

## AUTHOR CONTRIBUTIONS

All authors made substantial contributions to the conception or design of the work; or the acquisition, analysis or interpretation of data or the creation of new software used in the work. All authors drafted the work or revised it critically for important intellectual content. All authors approved the version to be published. All authors agree to be accountable for all aspects of the work in ensuring that questions related to the accuracy or integrity of any part of the work are appropriately investigated and resolved.

## CONFLICT OF INTEREST STATEMENT

The authors declare no conflicts of interest.

## ETHICS STATEMENT

Please include the name of the institutional review board (IRB) and the approval number. If not applicable, please state so. Not applicable – this study was IRB‐exempt at our institution.

## Data Availability

The data that support the findings of this study are available from the corresponding author upon reasonable request.
